# Female post-copulatory behavior in a group of olive baboons (*Papio anubis*) infected by *Treponema pallidum*

**DOI:** 10.1371/journal.pone.0261894

**Published:** 2022-01-20

**Authors:** Filipa M. D. Paciência, Idrissa S. Chuma, Iddi F. Lipende, Sascha Knauf, Dietmar Zinner

**Affiliations:** 1 Cognitive Ethology Laboratory, Deutsches Primatenzentrum, Leibniz Institute for Primate Research, Göttingen, Germany; 2 Work Group Neglected Tropical Diseases, Infection Biology Unit, Deutsches Primatenzentrum, Leibniz Institute for Primate Research, Göttingen, Germany; 3 Sokoine University of Agriculture, Chuo Kikuu, Morogoro, Tanzania; 4 Institute of International Animal Health, Friedrich-Loeffler-Institute, Federal Research Institute for Animal Health, Greifswald, Isle of Riems, Germany; 5 Department for Animal Sciences, Georg-August-University, Göttingen, Germany; 6 Leibniz Science Campus Primate Cognition, Göttingen, Germany; 7 Department of Primate Cognition, Georg-August-University, Göttingen, Germany; UiT Norges arktiske universitet, NORWAY

## Abstract

Pathogens exert a profound and pervasive cost on various aspects of primate sociality and reproduction. In olive baboons (*Papio anubis*) at Lake Manyara National Park, Tanzania, genital skin ulcers, caused by the bacterium *Treponema pallidum* subsp. *pertenue*, are associated with increased female mating avoidance and altered male mating patterns at a pre-copulatory and copulatory level. Beyond this, mating is also comprised of post-copulatory interactions among sexual partners (i.e., copulation calls, darting [post-copulatory sprint away from the male], and post-copulatory grooming). In baboons, female post-copulatory behavior is hypothesized to incite male-male competition, promote subsequent copulations, and/or strengthen the bonds between the mating pairs. Due to a higher reproductive burden (i.e. pregnancy, lactation, infant rearing), females should avoid proceptive behavior after mating to decrease further exposure to potential pathogens. To investigate whether the presence of genital skin ulcers has an impact at the post-copulatory level, we analyzed 517 copulation events of 33 cycling females and 29 males with and without genital skin ulcers. The occurrence of female post-copulatory behaviors was not altered by genital skin ulcerations in males. Similar to other baboon populations, females in our study group were more likely to utter copulation calls after an ejaculatory copulation. The likelihood of darting was higher after ejaculatory copulations and with the presence of copulation calls. Post-copulatory grooming (i.e., occurring within 15 seconds after a copulation) was not frequently observed. Our results indicate that despite the presence of conspicuous signs of disease, female post-copulatory behavior was not affected by the genital health status of the males. This indicates that in our study group, infection cues caused by *T*. *pallidum* subsp. *pertenue* play a major role before and during mating, but not after mating. The post-copulatory behavior of females is most likely affected by physiological or evolutionary constraints other than sexually transmitted infections.

## Introduction

An important fitness criterion in the mating context is the health status of the potential sexual partner. Individuals should choose healthy partners, since mating with a sick individual may not only have negative effects on the offspring (i.e. a poor health status can be an indication of a poor immune system which would then be passed on to the offspring) but also on the health and reproduction of the individual itself and the health of the offspring if the disease can be transmitted [1,2]. The latter becomes particularly obvious if the disease is sexually transmitted. Furthermore, poor health status can alter the individual´s attractiveness as a sexual partner, its competitive ability, and performance in the mating context [3].

In baboons, copulations are usually defined by male mounting with intromission and pelvic thrusts upon the female, which can culminate with ejaculation [4,5]. An ejaculatory mount is usually identified by an ejaculation pause, where the male remains rigid upon the female for a few seconds [4] and often a sperm plug is visible on the female’s external genitalia. During or after copulation, females may utter copulation calls, which are typically low-frequency rhythmic vocalizations [6]. Besides, female baboons often exhibit a characteristic post-coital sprint over several meters away from the male, a post-copulatory withdraw-behavior called ‘darting’ [4,5,7–11]. Finally, pairs may engage in post-copulatory grooming (PCG), which can be initiated either by the male or by the female [4].

Although post-copulatory behavior could play an important role in both female choice and sperm competition [12], little is known about the function of post-copulatory behavior in nonhuman primates. It has been suggested that the presence of these post-copulatory behaviors might (i) incite male-male competition by attracting non mate-guarding (i.e., non-consorting) males [13], (ii) encourage indirect sperm competition between males [14], (iii) increase the probability of subsequent matings with the same male [15,16], or (iv) strengthen female-male social relationships [17,18]. These hypotheses are, however, not mutually exclusive.

**Fig 1:**
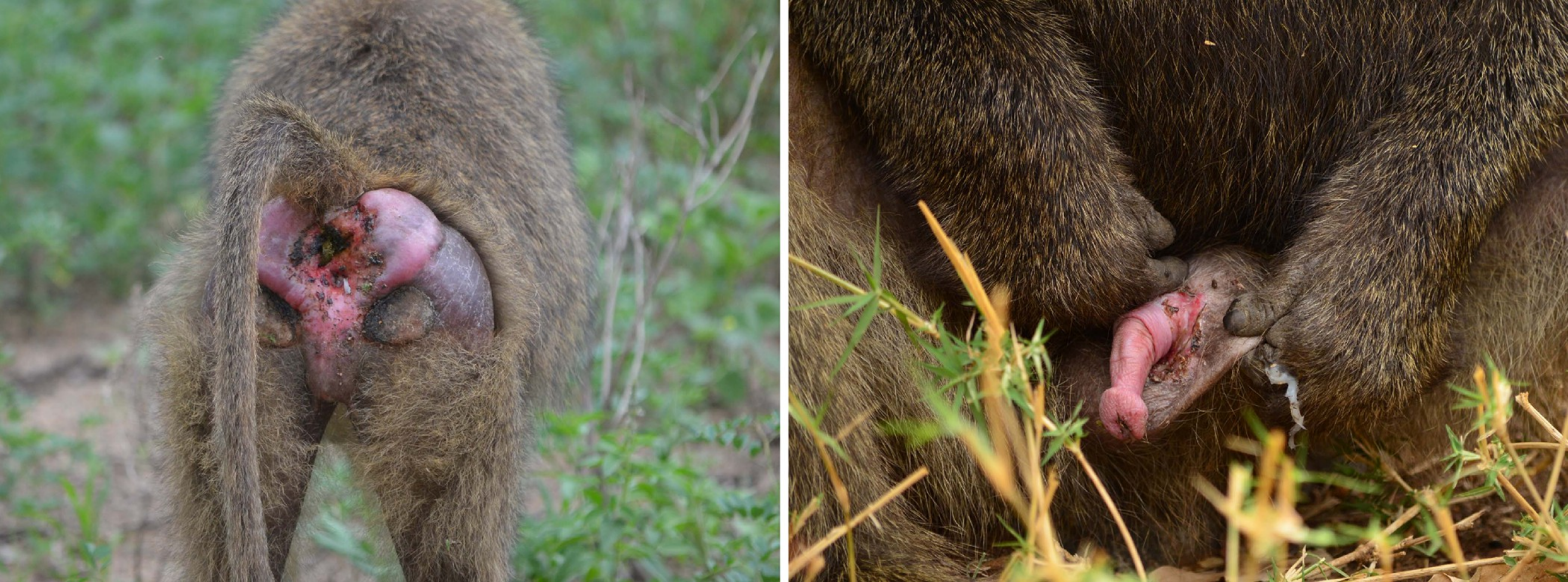
Genital skin ulcerations caused by *Treponema pallidum* subsp. pertenue. Olive baboon adult female (left) and adult male (right) at Lake Manyara National Park, Tanzania.

In Lake Manyara National Park (LMNP), Tanzania, olive baboons (*Papio anubis*) are infected with the bacterium *Treponema pallidum* subsp. *pertenue* as serological and genetic analyses indicated [19–22]. This bacterium also causes human yaws. In baboons, this infection is associated with moderate to severe skin ulcers of the anogenital area and external genitalia in both males and females (Fig 1). As observed in humans infected with *T*. *pallidum*, we assumed that baboons are mainly infectious when skin lesions or genital ulcers are present [23].

Progressive scarification of the tissue can lead to a permanently open state of the vagina and anus in females, while in males it is reported to cause phimosis (i.e. inability to retract the skin covering the glans of the penis) or loss of the corpus penis [19]. The high frequency of observed genital ulcerations in sexually mature individuals suggests that the pathogen is sexually transmitted [19,20,24].

The presence of genital skin ulcers in baboons causes changes in the copulatory behavior of males and females [25]. Symptomatic males decrease the number of pelvic thrusts (which might compromise the ability to ejaculate and sire offspring); while females avoid mating when they or their potential sexual partners are genitally ulcerated [25]. An increase of sexual investment by males towards proceptive females might lead to a higher likelihood of subsequent matings with the same male [4], which in turn, leads to greater exposure to potential pathogens. Because *T*. *pallidum* requires skin-to-skin contact to be transmitted, factors such as contact frequency and duration, are important features for transmission which occur during mating. Therefore, increasing the rate of copulations (i.e. consecutive mating), due to proceptive behavior by the females, will lead to higher exposure and consequently to a higher likelihood of infection. Thus, females should not only avoid copulation with diseased males but also avoid showing any proceptive behavior, such as copulation calls, darting, and post-copulatory grooming.

## Materials and methods

This research adhered to the rules and regulations of the Tanzanian and German laws. The Animal Welfare and Ethics Committee of the German Primate Center approved the entire study (document no. E11-18). This study was carried out in accordance with the Tanzania Wildlife Research Institute’s Guidelines for Conducting Wildlife Research and with permission from Tanzania National Parks (TNP/HQ/C.10/13). Additional research permission was granted by the Commission for Science and Technology in Tanzania (2016-115-NA-2014-228).

### Study site and subjects

Fieldwork was conducted at LMNP, Northern Tanzania, during two field seasons (April to December) in 2015 and 2016. Our baboon group was habituated within four months before the data collection phase of the study to facilitate behavioral observations from a distance of fewer than five meters. Our baboon study group consisted of approximately 170 individuals, with 53 adult and subadult females, 35 adult and subadult males, and more than 70 immatures. In our analyses, we included 33 cycling females and their 29 male partners, which could all be individually identified. The genital health status (GHS) was visually assessed and all adult and subadult individuals were classified as either genitally “ulcerated” or “non-ulcerated” using macroscopic visual cues [19]. Genital ulcerations could range from small-medium ulcers to severe mutilation of the outer genitalia (Fig 1). We defined age categories as published elsewhere [26]. Briefly, adult females were characterized by full body size, while sub-adult females were smaller and lacked elongated nipples (but were already cycling). Adult males were characterized by their large body size and fully developed secondary sexual traits (elongated canines and large shoulder mane), whereas subadult males were larger than adult females but lacked these secondary sexual characteristics.

### Behavioral data

We conducted focal follows from dawn to dusk, but distributed the focal periods among several females with peak swellings per day (total 33 cycling females) [27]. To maximize the number of observed mating events, we focused on females in their peak estrus, denoted by maximal tumescence and the bright pink color of their anogenital skin [28]. Due to the large group size, several females were in peak estrus simultaneously. Therefore, focal observations were performed opportunistically taking into account the proximity of the observer to the respective female, the visibility and the easiness of the individuals (i.e. due to recent habituation to human observers, some individuals were still shy). Thus, when following a female with an estrus swelling, we did not wait until remating occurred, but switched observations among females several times a day. We collected 597 hours of observation data, with an average of 16.40 ± 10.02 hours (mean ± SD, range 1.50–39.00 hours) per focal female. We collected data on the number of mating events, type of copulation, and the presence of copulation calls, darting behavior, and PCG (Table 1). Behavioral data were recorded in the field on a hand-held Samsung Galaxy using Pendragon 5.1.2 software (Pendragon Software Corporation, USA).

**Table 1:** Definition of behavioral variables.

	Definition
**Copulation/mating event**	male mounting an estrous female and performing pelvic thrusts (with intromission*) with or without ejaculation
**Type of copulation**	ejaculatory or non-ejaculatory: indicated by visible fresh sperm on the male’s penis or by the sperm plug on the female’s genitalia after copulation
**Pelvic thrusts**	number of male pelvic thrusts during copulation
**Copulation call**	context-specific calls females utter during or at the end of a copulation
**Darting**	rapid withdraw in which a female can run several meters away from the male after copulation
**Post-copulatory grooming (PCG)**	male/female grooms the mating partner 15 seconds after copulation occurred

*Except for severely ulcerated males that lack the corpus penis as intromission cannot occur.

### Statistical analysis

We ran generalized linear mixed models (GLMM, [29]) to examine the post-copulatory behavior of our baboon population. All models were performed in R v3.4.4 [30] with the lme4 package v 1.1–15 [31]. Maximum likelihood ratio tests were used to test the full model with fixed factors against the null model (i.e. without fixed factors) [32]. Potential collinearity issues using Variance Inflation factors [33,34] were checked but did not reveal any issues. Reported p-values were based on tests of the individual fixed effects using a likelihood ratio test (R function drop1 with argument *test* set to “Chisq”; [35]). We included the GHS of both sexes as fixed predictors and included male identity, female identity, and dyad identities as random factors. We also tested the interaction between GHS of males and females. Since this did not significantly improve any model fit, we excluded it from all our final models for parsimony and a more reliable interpretation of the main effects (the outcome of the models containing the interaction can be found as supplementary material). In all models, we included the female focal observation hours (log-transformed) as an offset term to control for observation effort [36]. Data and r-codes of the models can be found at [37].

### Model description

#### Model 1: Female copulation calls

The first model analyzed whether the occurrence of copulation calls was affected by the male and female GHS, the type of copulation, or the number of pelvic thrusts. The response variable was the presence or absence of copulation calls per mating event (1/0) with a binomial error structure and a logit link function.

#### Model 2: Female darting behavior

With the second model, we examined whether post-copulatory darting was affected by the male and female GHS, type of copulation, and the occurrence of copulation calls. The response variable was the presence or absence of darting per mating event (1/0) with a binomial error structure and a logit link function.

#### Model 3: Occurrence of post-copulatory grooming (PCG)

With the third model, we investigated whether the occurrence of PCG was affected by the male and female GHS, the presence of copulation calls, and the type of copulation. Here the response variable was the presence or absence of PCG per mating event (1/0) with a binomial error structure and a logit link function.

#### Model 4: Duration of post-copulatory grooming (PCG) by females

With this model, we examined whether the duration of PCG (in seconds) by females on males was affected by the presence of copulation calls and the type of copulation. The model assumed that the duration of PCG depended on the presence of copulation calls, type of copulation, and the male and female GHS. The model was fitted using the glmmADMB package [38] with a negative binomial error structure and a logit link function.

## Results

**Fig 2:**
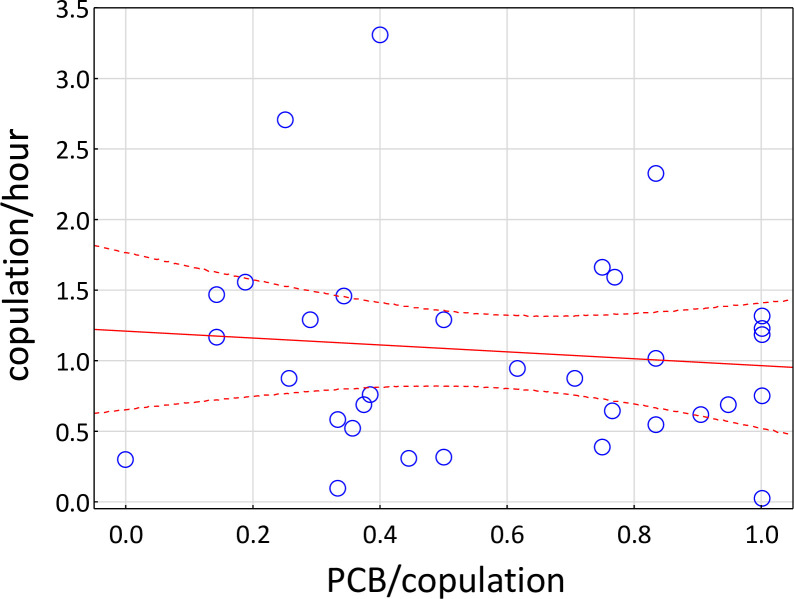
Frequency of copulation per female per hour by frequency of PCB (post-copulatory behavior) per female per copulation (blue circles represent individual females; r = -0.10; dashed lines = 95% confidence interval).

The prevalence of ‘genital ulcerated’ individuals in our study group (determined visually) remained relatively stable throughout the 18-months study period. Only three adult females and three adult males switched from ‘non-ulcerated’ to ‘ulcerated’ between field seasons. Therefore, at the end of the study, 44% (N = 23) of the 53 adult and subadult females and 47% (N = 17) of the 35 adult and subadult males displayed genital ulcers. Among individuals that participated in sexual interactions, genital ulcers were observed in 40% (N = 33) of the females and 53% (N = 35) of the males. In total, we observed 517 copulations among 33 females and 29 males. Ninety-five percent of the copulations were between females and their respective consort partners. In general, none of the post-copulatory behaviors led to an increase of the (re-)mating frequency. The number of copulations per female per hour was not affected by the occurrence and frequency of post-copulatory behaviors (Fig 2).

**Fig 3:**
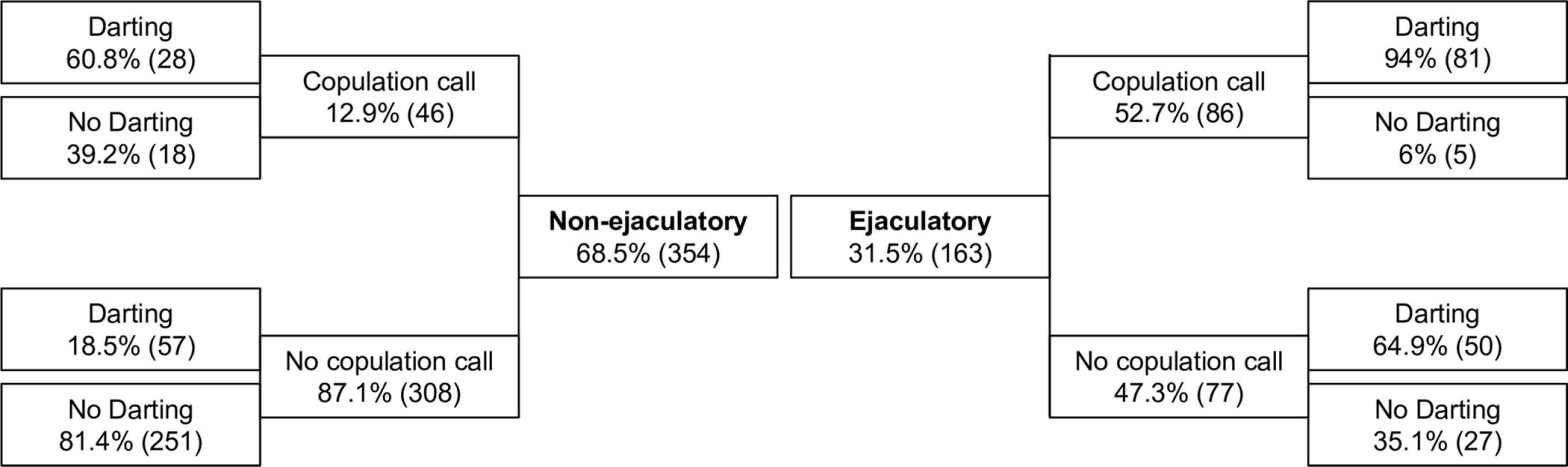
Frequency of copulation calls and darting. Frequencies are divided between ejaculatory copulations (right; N = 163) and non-ejaculatory copulations (left; N = 354). Total number of copulations (N = 517).

Evidence for ejaculation was found in 31.5% (N = 163) of the copulations. Females uttered copulation calls in 25.5% (N = 132), darting occurred in 41.7% (N = 216) and PCG in 27.2% (N = 141). The frequency of copulation calls and darting differed slightly between ejaculatory to non-ejaculatory copulations (Fig 3).

In this study, males who lacked the corpus penis were still able to ejaculate. These males would ejaculate towards the ground or against their legs as there was no way to direct the sperm into the female´s genital tract. Nevertheless, two females in our study group uttered copulation calls even when mating with males lacking the corpus penis, where intromission was not observed (i.e., males solely performed pelvic thrusts). The likelihood of uttering copulation calls increased with ejaculatory mating (p<0.001, Table 2), but was not affected by the GHS of either males or females. Adding an interaction between the male and female GHS was not a significant predictor for the likelihood of uttering copulation calls (p>0.05, S1 Table Supplementary Material).

**Table 2:** Female copulation call model. Binary GLMM evaluating if the likelihood of uttering a copulation call was affected by the male and female GHS, and the type of copulation (ejaculatory vs non-ejaculatory).

Term	Estimate	SE	CI lower	CI upper	z value	P
Intercept	-3.725	0.830	-5.639	-2.245	-4.490	-
Male GHS	0.057	0.803	-1.518	1.758	0.071	0.944
Female GHS	1.482	0.945	-0.347	3.553	1.569	0.117
Type of copulation	2.800	0.430	2.005	3.706	5.759	<0.001

Estimates, standard errors (SE), z-values, and 2.5% and 97.5% confidence intervals (CI) are shown for fixed effects. Intercept with a reference category is shown for ulcerated individuals and ejaculatory events.

Darting occurred after 80% (N = 131) of the copulations with ejaculation, but only after 24% (N = 85) of the non-ejaculatory copulations. Darting was also more frequent when females uttered copulation calls (94%, N = 81). The likelihood of darting was higher when females gave copulation calls and when the male ejaculated (p<0.001, Table 3), but was not affected by the GHS of either males or females. Adding an interaction between the male and female GHS was not a significant predictor for the likelihood of female darting (p>0.05, S2 Table Supplementary Material).

**Table 3:** Female post-copulatory darting model. Binomial GLMM evaluating if the likelihood of female darting was influenced by the male and female GHS, presence of copulation calls, and type of copulation (ejaculatory vs non-ejaculatory).

Term	Estimate	SE	CI lower	CI upper	z value	P
Intercept	-1.468	0.503	-2.537	-0.497	-2.919	-
Male GHS	-0.525	0.621	-1.714	0.770	-0.844	0.118
Female GHS	0.883	0.565	-0.242	2.092	1.563	0.398
Copulation call	2.408	0.480	1.502	3.402	5.017	<0.001
Type of copulation	2.500	0.380	1.781	3.277	6.586	<0.001

Estimates, standard errors (SE), z-values, and 2.5% and 97.5% confidence intervals (CI) are shown for fixed effects. Intercept with reference category is shown for ulcerated individuals, presence of copulation calls, and ejaculatory events.

Darting never led to the termination of a consortship as males kept track of the females, even if the female covered distances of more than 10 meters. Moreover, darting also seems not to increase the likelihood of a consort take-over, because consort take-overs occurred rarely in our group (N = 7) in the 18-month study period. The occurrence of PCG was also not affected by the GHS of either males or females and also not by the occurrence of copulation calls or by the type of copulation (Table 4). Adding an interaction between the male and female GHS was not a significant predictor for the likelihood of performing PCG (p>0.05, S3 Table Supplementary Material).

**Table 4:** Post-copulatory grooming (PCG)-presence model. Binomial GLMM evaluating if the likelihood of PCG was affected by the male and female GHS, presence of copulation calls, and type of copulation (ejaculatory vs. non-ejaculatory).

Term	Estimate	SE	CI lower	CI upper	z value	P
Intercept	-1.278	0.257	-1.819	-0.770	-4.978	-
Male GHS	0.520	0.307	-0.125	1.130	1.695	0.090
Female GHS	0.017	0.377	-0.828	0.681	-0.045	0.964
Copulation call	-0.070	0.319	-0.707	0.561	-0.220	0.826
Type of copulation	-0.178	0.278	-0.739	0.362	-0.641	0.521

Estimates, standard errors (SE), z-values, and 2.5% and 97.5% confidence intervals (CI) are shown for fixed effects. Intercept with reference category is shown for ulcerated individuals, presence of copulation calls, and ejaculatory events.

In most cases (72.8%, N = 376), no PCG occurred. When it occurred, males initiated PCG more often than females regardless of the type of copulation (Table 5).

**Table 5:** Frequency of post-copulatory grooming (PCG) initiation in relation to copulation type (number of cases in parentheses).

	Copulations (512)*	Ejaculatory 30.6% (159)	Non-ejaculatory 68.2% (353)
No PCG	72.7% (376)	77.3% (123)	71.5% (253)
Male initiated	17.7% (92)	15.0% (24)	19.2% (68)
Female initiated	8.5% (44)	4.5% (12)	9.0% (32)

*Five copulations were excluded from the total (N = 517) as PCG could not be assessed properly.

Similar to the occurrence of PCG, also the duration of PCG was not affected by the GHS of either males or females (Table 6). However, the duration of PCG performed by males was longer when females uttered copulation calls (p = 0.019). The same effect was not found for PCG initiated by females (Table 6). Adding an interaction between the GHS of males and females was not a significant predictor for the duration of female PCG (p>0.05, S4 Table Supplementary Material).

**Table 6:** PCG duration model by females. GLMMs evaluating if the duration of female PCG was affected by the presence of copulation calls and type of copulation (ejaculatory vs non-ejaculatory).

Term	Estimate	SE	CI lower	CI upper	z value	P
Intercept	5.229	0.229	4.780	5.677	22.84	-
Male GHS	0.337	0.327	-0.304	0.978	1.029	0.304
Female GHS	-0.124	0.331	-0.773	0.525	-0.375	0.708
Copulation call	-0.147	0.456	-0.485	0.780	0.456	0.648
Type of copulation	0.164	0.588	-0.381	0.709	0.588	0.556

Estimates, standard errors (SE), z-values, and 2.5% and 97.5% confidence intervals (CI) are shown for fixed effects. Intercept with reference category is shown for ulcerated individuals, presence of copulation calls, and ejaculatory events.

## Discussion

In contrast to our main prediction, infection cues do not lead to a decrease in the frequency and duration of post-copulatory behaviors of females, and thus these behaviors cannot be regarded as a further means to minimize copulation frequency with infected males. Instead, our results show that, after a copulation, females behaved similarly to females of other baboon populations. Their post-copulatory behavior was mainly affected by the type of copulation, i.e., whether an ejaculation occurred or not. As in other baboon populations, ejaculatory matings lead to a higher frequency of copulation calls [4,10,15,16,18,39] but see [40], and female darting [16]. Copulation calls and darting are usually tightly linked and might facilitate bonding with particular males, to reduce harassment and coercion by other males [8,41]. Regarding post-copulatory grooming, quantitative studies are scarce for primates. In Barbary macaques, this behavior occurs in half of the mating events [42–45]. In this species, males are more likely to groom females after ejaculatory copulations, while females groom males more often after non-ejaculatory matings [45]. This contrasts with the results of our study, where PCG was not observed in the majority of the cases, and, if it occurred, it was neither affected by the type of copulation nor by the presence of copulation calls. It also did not increase the frequency of re-mating (Fig 2).

In olive baboons, females have been described to run immediately from the mating partner towards another male after copulation [7], leading to consort take-overs [8]. Such behavior was not observed at LMNP as the darting female was usually followed by the mate-guarding male, and consort take-overs were seldom observed. This might be due to the long-term bonds observed between males and females in estrus, as females would frequently mate with the same male during different cycling periods [25].

Regarding the function of PCB, we did not find support for hypotheses (i) ‘inciting male-male competition by attracting non mate-guarding males’, (ii) encourage indirect sperm competition between males, or (iii) increase the probability of subsequent matings with the same male. Consort takeovers and copulations outside of consorts were very rare and PCB also did not increase to probability of further copulations with respective consort partners. We therefore think, that PCB is used as a means to strengthen female-male social relationships (hypothesis iv).

*Treponema pallidum* infection cues do not influence the occurrence and outcome of post-copulatory behavior in our study females. We suggest that either the disease is too young in evolutionary terms, and there was not enough time for species to evolve behavioral adaptations, or the cost-benefit ratio in terms of fitness, by supporting the establishment and maintenance of stable female-male bonds. In large groups with a high number of (unfamiliar) males, risks of sexual coercion and infanticide might be particularly high [46] and thus, as pathogen exposure might have already occurred during copulation, it could be more advantageous for females to keep investing in reliable bonds with their mating partners. In our study, the lack of post-copulatory behavioral avoidance, together with the long-term bonds observed between individuals, could mean that females use post-copulatory behavior as a way to strengthen the bonds with the current (long-term) mating partner.

In general, post-copulatory behavior is still poorly understood in nonhuman primates, even more so in combination with infectious diseases. Therefore, we suggest that the post-copulatory behavior of our study females is most likely more impacted by physiological or evolutionary constraints other than sexually transmitted infections.

## Supporting information

S1 Table Female copulation call interaction model. Binary GLMM evaluating if the likelihood of uttering a copulation call is affected by the male and female GHS and the type of copulation. Estimates, standard errors (SE), z-values, and 2.5% and 97.5% confidence intervals (CI) are shown for fixed effects. Intercept with a reference category for ulcerated individuals and ejaculatory events.
(DOCX)

S2 Table Female darting behavior interaction model. Binomial GLMM evaluating if the likelihood of darting is influenced by the male and female GHS, presence of copulation calls and type of copulation. Estimates, standard errors (SE), z-values, and 2.5% and 97.5% confidence intervals (CI) are shown for fixed effects. Intercept with reference category for ulcerated individuals, presence of copulation calls and ejaculatory events.
(DOCX)

S3 Table Presence of post-copulatory grooming interaction model. Binomial GLMM evaluating if the likelihood of PCG is affected by the male and female GHS, presence of copulation calls and type of copulation. Estimates, standard errors (SE), z-values, and 2.5% and 97.5% confidence intervals (CI) are shown for fixed effects. Intercept with reference category for ulcerated individuals, presence of copulation calls and ejaculatory events.
(DOCX)

S4 Table PCG duration interaction model by females. GLMMs evaluating if the duration of female PCG is affected by the by the male and female GHS, presence of copulation calls and type of copulation. Estimates, standard errors (SE), z-values, and 2.5% and 97.5% confidence intervals (CI) are shown for fixed effects. Intercept with reference category for ulcerated individuals, presence of copulation calls and ejaculatory events.
(DOCX)

## References

[1] Hillgarth N. Ectoparasite transfer during mating in ring-necked pheasants *Phasianus colchicus*. J Avian Biol. 1996; 27(3): 260–262. 10.2307/3677232.

[2] Martinez-Padilla J, Vergara P, Mougeot F, Redpath SM. Parasitized mates increase infection risk for partners. Am Nat. 2012; 179 (6): 811–820. doi: 10.1086/665664 22617268

[3] Beltran-Bech S, Richard FJ. Impact of infection on mate choice. Anim Behav. 2014; 90(0): 159–170. 10.1016/j.anbehav.2014.01.026.

[4] Saayman GS. The menstrual cycle and sexual behavior in a troop of free-living chacma baboons (*Papio ursinus*). Folia Primatol. 1970; 12 (2): 81–110. 10.1159/000155283. 4984817

[5] Ransom TW. Beach Troop of the Gombe. Lewisburg: Bucknell University Press. 1981.

[6] Bouquet Y, Stephan C, Johnson CA, Rothman JM, Neumann C, Zuberbühler K. Comparing functions of copulation calls in wild olive baboons, *Papio anubis*, using multimodel inference. Anim Behav. 2018; 135: 187–197. 10.1016/j.anbehav.2017.11.019.

[7] Hall KRL, DeVore I. Baboon social behavior. In DeVore I editor. Primate Behavior: Field Studies of Monkeys and Apes (pp. 53–110). New York: Holt, Rinehart, and Winston; 1965.

[8] Smuts BB. Sex and Friendship in Baboons. New York: Aldine. 1985.

[9] Bercovitch FB. Female cooperation, consortship maintenance, and male mating success in savanna baboons. Anim Behav. 1995; 50(1): 137–149. 10.1006/anbe.1995.0227.

[10] Collins DA. Behaviour and Patterns of Mating among Adult Yellow Baboons (*Papio cynocephalus*). PhD Thesis, University of Edinburgh. 1981.

[11] O’Connell SM, Cowlishaw G. The post-copulation withdrawal response in female baboons: a functional-analysis. Primates. 1995; 36(3): 441–446. 10.1007/BF02382866.

[12] Birkhead TR, Pizzari T. Postcopulatory sexual selection. Nat Rev Genet. 2002; 3(4): 262–273. doi: 10.1038/nrg774 11967551

[13] Oda R, Masataka N. Functional significance of female Japanese macaque copulatory calls. Folia Primatol. 1992; 58 (3): 146–149. doi: 10.1159/000156621 1398344

[14] Keller L, Reeve HK. Why do females mate with multiple males? The sexually selected sperm hypothesis. Adv Study Behav. 1995; 24: 291e315. 10.1016/s0065-3454(08)60397-6.

[15] O’Connell SM, Cowlishaw G. Infanticide avoidance, sperm competition and mate choice: the function of copulation calls in female baboons. Anim Behav. 1994; 48(3): 687–694. 10.1006/anbe.1994.1288.

[16] Walz JT. Competition, Coercion, and Choice: The Sex Lives of Female Olive Baboons (*Papio anubis*). PhD Thesis. Ohio State University; 2016.

[17] Seyfarth RM. Social relationships among adult male and female baboons, I. Behaviour during sexual consortship. Behaviour. 1978; 64:204–226.

[18] Todt D, Hammerschmidt K, Ansorge V, Fischer J. The vocal behavior of Barbary macaques (*Macaca sylvanus*): call features and their performance in infants and adults. In: Zimmermann E, Newman JD, Juergens U editors. Current Topics in Primate Vocal Communications. New York: Plenum Press; 1995. pp. 141–160.

[19] Knauf S, Batamuzi EK, Mlengeya T, Kilewo M, Lejora IAV, Nordhoff M, et al. Treponema infection associated with genital ulceration in wild baboons. Vet Pathol. 2012; 49(2): 292–303. doi: 10.1177/0300985811402839 21411621

[20] Knauf S, Gogarten JF, Schuenemann VJ, De Nys HM, Düx A, Strouhal M, et al. Nonhuman primates across sub-Saharan Africa are infected with the yaws bacterium Treponema pallidum subsp. pertenue. Emerg Microbes Infect. 2018; 7(1): 1–4. doi: 10.1038/s41426-017-0002-0 30228266 PMC6143531

[21] Harper KN, Fyumagwa RD, Hoare R, Wambura PN, Coppenhaver DH, Sapolsky R, et al. Treponema pallidum infection in the wild baboons of East Africa: distribution and genetic characterization of the strains responsible. PLoS ONE. 2012; 7(12): e50882. doi: 10.1371/journal.pone.0050882 23284649 PMC3527465

[22] Chuma IS, Batamuzi EK, Collins DA, Fyumagwa RD, Hallmaier-Wacker LK, Kazwala RR, et al. Widespread Treponema pallidum infection in nonhuman primates, Tanzania. Emerg Infec Dis. 2016; 24(6): 1002–1009. 10.3201/eid2406.180037. PMC6004850 29774840

[23] Holmes KK, Sparling PF, Stamm WE, Piot P, Wasserheit JN, Corey L, et al. Sexually Transmitted Diseases. 4^th^ ed. New York: McGraw Hill Professional; 2007.

[24] Mlengeya TDK. Distribution Pattern of a Sexually Transmitted Disease (STD) of Olive Baboon in Lake Manyara National Park, Tanzania. MsC Thesis; College of African Wildlife Management. 2004.

[25] Paciência FMD, Rushmore J, Chuma IS, Lipende IF, Caillaud D, Knauf S, et al. Mating avoidance in female olive baboons (*Papio anubis*) infected by *Treponema pallidum*. Sci Adv. 2019; 5(12): eaaw9724. doi: 10.1126/sciadv.aaw9724 31840059 PMC6892622

[26] Rowell T. Long-term changes in a population of Ugandan baboons. Folia Primatol. 1969; 11(4): 241–254. 10.1159/000155273. 4982884

[27] Altmann J. Observational study of behavior: Sampling methods. Behaviour 1974; 49, 227–267. doi: 10.1163/156853974x00534 4597405

[28] Zinner D, Nunn CL, van Schaik CP, Kappeler PM. Sexual selection and exaggerated sexual swellings of female primates. In: Kappeler PM, van Schaik CP editors. Sexual Selection in Primates. New and Comparative Perspectives. Cambridge: Cambridge University Press. 2004. pp 71–84.

[29] Baayen RH. Analyzing Linguistic Data: A Practical Introduction to Statistics Using R. New York: Cambridge University Press; 2008.

[30] R Core Team R: A language and environment for statistical computing. R Foundation for Statistical Computing, Vienna, Austria. 2018.

[31] Bates D, Maechler M, Bolker B, Walker S. Fitting linear mixed-effects models using lme4. J Stat Softw. 2015; 67:1–48. 10.18637/jss.v067.i01.

[32] Faraway JJ. Extending Linear Models with R. Boca Raton: Chapman & Hall/CRC; 2006. doi: 10.1093/bioinformatics/btl127

[33] Quinn GP, Keough MJ. Experimental Designs and Data Analysis for Biologists. Cambridge: Cambridge University Press; 2002.

[34] Field A. Discovering Statistics using SPSS. London: Sage Publications; 2005.

[35] Barr DJ, Levy R, Scheepers C, Tily HJ. Random effects structure for confirmatory hypothesis testing: Keep it maximal. J Mem Lang. 2013; 68(3): 255–278. doi: 10.1016/j.jml.2012.11.001 24403724 PMC3881361

[36] McCullagh P, Nelder JA. Generalized linear models. 2nd edition. Chapman and Hall. London; 1989.

[37] Zinner D. Data and codes for: Female post-copulatory behavior in a group of olive baboons (*Papio anubis*) infected by *Treponema pallidum*. Göttingen Research Online / Data, 2021. 10.25625/H06NWS. PMC8775205 35051197

[38] Fournier DA, Skaug HJ, Ancheta J, Ianelli J, Magnusson A, Maunder M, et al. AD Model Builder: using automatic differentiation for statistical inference of highly parameterized complex nonlinear models. Optim Methods Softw. 2021; 27(2): 233–249. 10.1080/10556788.2011.597854.

[39] Deputte BL, Goustard M. Copulatory vocalizations of female macaques (*Macaca fascicularis*): variability factors analysis. Primates. 1980; 21(1): 83–99. 10.1007/BF02383826.

[40] Semple S, McComb K, Alberts S, Altmann J. Information content of female copulation calls in yellow baboons. Am J Primatol. 2002; 56(1): 43–56. doi: 10.1002/ajp.1062 11793412

[41] Palombit RA. "Friendship" with males: a female counterstrategy to infanticide in chacma baboons of the Okavango Delta. In: Muller M. N. & Wrangham R. W. (Eds.), *Sexual Coercion in Primates and Humans: An Evolutionary Perspective on Male Aggression Against Females*. Cambridge, MA: Harvard University Press. 2009. pp 377–409. doi: 10.1007/s00265-009-0781-y

[42] Taub DM. Female choice and mating strategies among wild Barbary macaques (*Macaca sylvanus* L). In: Lindburg DG editor. The Macaques: Studies in Ecology, Behavior and Evolution. New York: Van Nostrand Reinhold. 1980. pp 287–344.

[43] Small MF. Promiscuity in Barbary macaques (*Macaca sylvanus*). Am J Primatol. 1990; 20(4): 267–282. doi: 10.1002/ajp.1350200403 32075348

[44] Kuester J, Paul A. Influence of male competition and female mate choice on male mating success in Barbary macaques (*Macaca sylvanus*). Behaviour. 1992; 120(3–4): 192–217. 10.1163/156853992X00606.

[45] Sonnweber RS, Massen JJM, Fitch WT. Post-copulatory grooming: a conditional mating strategy? Behav Ecol Sociobiol. 2015; 69(11): 1749–1759. 10.1007/s00265-015-1987-9.

[46] van Schaik CP. Social evolution in primates: The role of ecological factors and male behaviour. In: Runciman WG, Maynard Smith J, Dunbar RIM editors. Evolution of Social Behaviour Patterns in Primates and Man. Oxford: Oxford University Press. 1996. pp. 9–31.

