# Supplementary material for: Female post-copulatory behavior in a group of olive baboons (Papio anubis) infected by Treponema pallidum
Source: PLoS One. 2022 Jan 20;17(1):e0261894. doi: 10.1371/journal.pone.0261894 (PMC8775205; doi:10.1371/journal.pone.0261894)
Supplement: S1 Table — Binary GLMM evaluating if the likelihood of uttering a copulation call is affected by the male and female GHS and the type of copulation. Estimates, standard errors (SE), z-values, and 2.5% and 97.5% confidence intervals (CI) are shown for fixed effects. Intercept with a reference category for ulcerated individuals and ejaculatory events. (DOCX) [file pone.0261894.s001.docx]

**Supporting Information**

**TABLE S1. Female copulation call interaction model.** Binary GLMM evaluating if the likelihood of uttering a copulation call is affected by the male and female GHS and the type of copulation. Estimates, standard errors (SE), z-values, and 2.5% and 97.5% confidence intervals (CI) are shown for fixed effects. Intercept with a reference category for ulcerated individuals and ejaculatory events.

|  | Estimate | SE | CI lower | CI upper | z value | Pr(>\|z\|) |
| --- | --- | --- | --- | --- | --- | --- |
| (Intercept) | -3.618 | 0.842 | -5.837 | -2.093 | -4.296 | - |
| Female GHS | 1.121 | 1.185 | -1.279 | 3.771 | 0.946 | 0.344 |
| Male GHS | -0.202 | 0.959 | -2.369 | 1.733 | -0.211 | 0.833 |
| Type of copulation | 2.815 | 0.432 | 2.037 | 4.010 | 6.511 | 0.000 |
| Female GHS: Male GHS | 0.732 | 1.500 | -2.146 | 3.684 | 0.488 | 0.626 |
