# Supplementary material for: Female post-copulatory behavior in a group of olive baboons (Papio anubis) infected by Treponema pallidum
Source: PLoS One. 2022 Jan 20;17(1):e0261894. doi: 10.1371/journal.pone.0261894 (PMC8775205; doi:10.1371/journal.pone.0261894)
Supplement: S2 Table — Binomial GLMM evaluating if the likelihood of darting is influenced by the male and female GHS, presence of copulation calls and type of copulation. Estimates, standard errors (SE), z-values, and 2.5% and 97.5% confidence intervals (CI) are shown for fixed effects. Intercept with reference category for ulcerated individuals, presence of copulation calls and ejaculatory events. (DOCX) [file pone.0261894.s002.docx]

**TABLE S2. Female darting behavior interaction model**. Binomial GLMM evaluating if the likelihood of darting is influenced by the male and female GHS, presence of copulation calls and type of copulation. Estimates, standard errors (SE), z-values, and 2.5% and 97.5% confidence intervals (CI) are shown for fixed effects. Intercept with reference category for ulcerated individuals, presence of copulation calls and ejaculatory events.

|  | Estimate | SE | CI lower | CI upper | z value | Pr(>\|z\|) |
| --- | --- | --- | --- | --- | --- | --- |
| (Intercept) | -1.603 | 0.534 | -2.691 | -0.601 | -3.004 | - |
| Female GHS | 1.439 | 0.820 | -0.238 | 3.225 | 1.755 | 0.079 |
| Male GHS | -0.202 | 0.712 | -1.611 | 1.284 | -0.283 | 0.777 |
| Copulation call | 2.450 | 0.490 | 1.561 | 3.532 | 5.000 | 0.000 |
| Type of copulation | 2.484 | 0.382 | 1.820 | 3.366 | 6.497 | 0.000 |
| Female GHS: Male GHS | -1.033 | 1.049 | -3.260 | 1.102 | -0.984 | 0.325 |
