# Supplementary material for: Female post-copulatory behavior in a group of olive baboons (Papio anubis) infected by Treponema pallidum
Source: PLoS One. 2022 Jan 20;17(1):e0261894. doi: 10.1371/journal.pone.0261894 (PMC8775205; doi:10.1371/journal.pone.0261894)
Supplement: S4 Table — GLMMs evaluating if the duration of female PCG is affected by the by the male and female GHS, presence of copulation calls and type of copulation. Estimates, standard errors (SE), z-values, and 2.5% and 97.5% confidence intervals (CI) are shown for fixed effects. Intercept with reference category for ulcerated individuals, presence of copulation calls and ejaculatory events. (DOCX) [file pone.0261894.s004.docx]

**TABLE S4.** **PCG duration interaction model by females.** GLMMs evaluating if the duration of female PCG is affected by the by the male and female GHS, presence of copulation calls and type of copulation. Estimates, standard errors (SE), z-values, and 2.5% and 97.5% confidence intervals (CI) are shown for fixed effects. Intercept with reference category for ulcerated individuals, presence of copulation calls and ejaculatory events.

|  | Estimate | SE | CI lower | CI upper | z value | Pr(>\|z\|) |
| --- | --- | --- | --- | --- | --- | --- |
| (Intercept) | -3.951 | 0.270 | 4.799 | 5.728 | -14.608 | - |
| Female GHS | -0.220 | 0.509 | -1.050 | 0.540 | -0.432 | 0.666 |
| Male GHS | 0.409 | 0.360 | -0.515 | 0.973 | 1.134 | 0.257 |
| Copulation call | -0.090 | 0.316 | -0.617 | 0.757 | -0.284 | 0.776 |
| Type of copulation | -0.157 | 0.279 | -0.355 | 0.772 | -0.562 | 0.574 |
| Female GHS: Male GHS | 0.034 | 0.655 | -0.997 | 1.777 | 0.052 | 0.959 |
